# Analysis of cis-regulatory changes underlying phenotype divergence shaped by domestication in pigs

**DOI:** 10.3389/fgene.2024.1421859

**Published:** 2024-11-08

**Authors:** Chunpeng Liu, Na Ao, Yuwen Liang, Tingting Ma, Qishan Wang, Zhen Wang, Fen Wu, Zhenyang Zhang, Yifei Fang, Minghui Wang, Yuchun Pan, Jing Fu

**Affiliations:** ^1^ College of Animal Science and Technology, Zhongkai University of Agriculture and Engineering, Guangzhou, China; ^2^ Innovative Institute of Animal Healthy Breeding, Zhongkai University of Agriculture and Engineering, Guangzhou, China; ^3^ College of Animal Sciences, Zhejiang University, Hangzhou, Zhejiang, China; ^4^ Department of Animal Science, Cornell University, Ithaca, NY, United States; ^5^ Center for Life Science Ventures, Cornell University, Ithaca, NY, United States

**Keywords:** ATAC-seq, chromatin accessibility, cis-regulatory element, allelic frequency, pig

## Abstract

**Background:**

Cis-regulatory elements (CREs) are regions of DNA that regulate the expression of nearby genes. Changes in these elements can lead to phenotypic variations and adaptations in different populations. However, the regulatory dynamics underlying the local adaptation of traits remain poorly understood in Chinese and Western pigs. By comparing the chromatin accessibility profiles of skeletal muscle, liver, and fat between these two pig populations, we aimed to identify key regulatory elements that could explain phenotypic differences observed between the two groups.

**Results:**

Our results revealed that the genome-wide chromatin accessibility profiles were largely similar at a qualitative level within tissues. However, we also identified local regions that exhibited quantitative differences, most of which occurred in liver tissue. Interestingly, we found that most of the increased chromatin accessibility in the livers of Chinese pigs was associated with tissue-specific openness. Furthermore, we observed a positive correlation between the ATAC-seq signal at the transcript start site (TSS) and the expression levels of nearby genes. Motif enrichment analysis revealed *NR2F1* as a key regulator in Chinese pigs. Differentially expressed genes (DEGs) in Chinese pigs showed enrichment for *NR2F1* response targets. One of the genes regulated by *NR2F1* in Chinese pigs, *NPC1*, harbored a high alternative allelic frequency in the intron region.

**Conclusion:**

Overall, our study provides valuable insights into the regulatory dynamics underlying phenotypic variation in pigs. By elucidating the role of CREs in driving phenotypic variation, we can better understand the genetic basis of complex traits and potentially identify targets for genetic improvement in livestock breeding programs.

## Background

The domestication of pigs likely began over 10,000 years ago in East Asia and Western Eurasia (Larson et al., 2005; Larson et al., 2010). Pigs have become the primary meat source and have served as biomedical models for human diseases and developmental processes in China (Joan K. et al., 2021). China’s diverse climate and geography have fostered the development of over 100 indigenous pig breeds, which are crucial for maintaining genetic diversity and are listed in the National Genetic Resources Protection catalog. The domestication process has significantly influenced phenotypic traits such as high reproductive capacity, robust tolerance to roughage, superior meat quality, strong disease resistance, and excellent maternal instincts (Mirte et al., 2014). In contrast, Western pig breeds are known for their rapid growth, high lean meat percentage, and efficient feed conversion, but they often lack robustness in other traits. These distinctions highlight the presence of favorable gene sets shaped during domestication. Indigenous pigs in China exhibit high allelic diversity (Tong et al., 2020; Zhang et al., 2018), suggesting that they may possess valuable genetic material for enhancing commercial breeds through crossbreeding. However, the molecular mechanisms underlying the diverse locally adaptive events remain poorly understood, posing challenges for future genetic improvements in these traits. Understanding the genetic basis of these differences, particularly the consistent divergence between Chinese and Western pigs, is crucial for understanding local adaptations and selective pressures.

Genome-wide association studies (GWAS) and quantitative trait loci (QTL) mapping are valuable tools for uncovering the genetic markers associated with phenotypic variation. The pig QTL database reports 48,875 QTLs for various traits (https://www.animalgenome.org/cgi-bin/QTLdb/SS/summary, release 28 April 2024), elucidating the genomic architecture of complex traits (Schaid et al., 2018). However, identifying functional candidate genes remains challenging, primarily because of its time-consuming and labor-intensive nature. Causal variants often reside in non-coding regions, complicating functional explanations. Mutations in promoter or enhancer regions will alter gene expression dynamics through the modulation of transcription factor (TF) binding (Cherry et al., 2020). Moreover, identifying causal variants is challenging, particularly in small, intensively selected livestock populations, due to linkage disequilibrium (LD). GWAS faces further challenges in interpreting associations due to unknown functional variants, genes, and cell types (Hannah et al., 2021). These variants likely collaborate to influence protein binding, regulatory changes, and functional module alterations. Therefore, elucidating the mechanisms of functional genes and causal variants, especially those involved in dynamic changes of regulatory networks through CREs, will help bridge the genotype–phenotype gap in the post-GWAS era.

Expression QTL (eQTL) studies provide valuable insights into the molecular mechanisms underlying cis-acting or trans-acting regulatory processes (Carmelo and Kadarmideen, 2020; Lourdes et al., 2020; Yan et al., 2020). However, understanding how these variants influence spatiotemporal expression across multiple layers, including transcription factor binding, regulatory signal processing, and chromatin state changes, remains unclear. Integrating epigenomic profiles from ATAC-seq and histone modifications with RNA-seq has emerged as a powerful approach to unravel the complex interplay between DNA activity, regulatory dynamics, and gene expression. Recent publications on genome-wide open chromatin landscapes in pigs (Pan et al., 2021; Zhao et al., 2021) have highlighted the roles of promoters and enhancers in complex traits and adaptive evolution. Nevertheless, it is still unclear what source of variation in CREs between Chinese and Western pigs contributes to significant differences.

In this study, we employed a thorough epigenomic approach to identify divergent CREs between Chinese and Western pigs, intending to annotate chromatin changes linked to phenotypic distinctions. Our analysis of chromatin accessibility across various tissues revealed substantial variations, particularly in liver tissue, where hundreds of CREs displayed distinct epigenetic profiles between the two pig populations. For example, examination of a specific CRE associated with *NPC1* (NPC intracellular cholesterol transporter 1), a gene crucial for cholesterol uptake and biosynthesis, revealed higher alternative allele frequencies at the binding site of *NR2F1* in Chinese pigs. This research enhances our understanding of how the divergence of CREs shaped by domestication influences phenotypic adaptation in pigs.

## Results

### Characterizing chromatin accessibility across tissues

**FIGURE 1:**
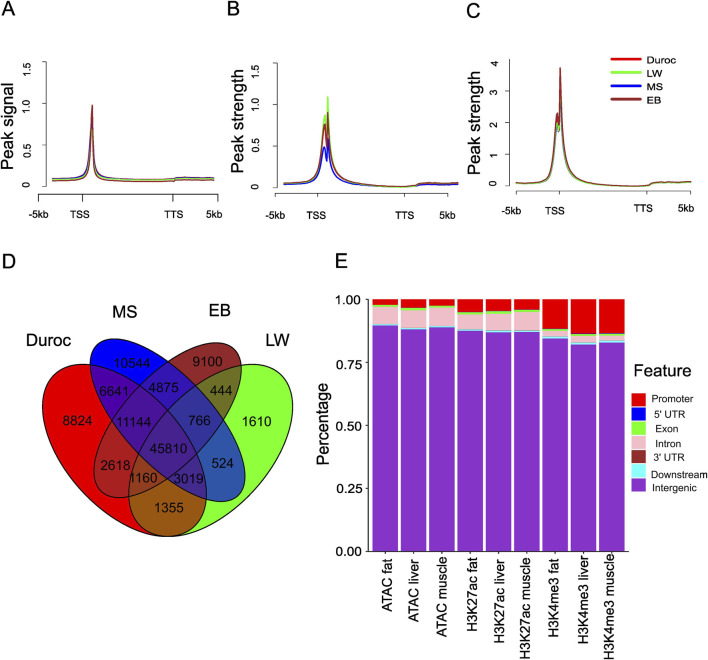
Genome-wide mapping of ATAC-seq, H3K27ac, and H3K4me3 sites across different pig breeds. **(A)** Metaplots of liver ATAC-seq signal in the genic region and the peak signal defined as the normalized value per million. **(B)** Metaplots of the liver H3K27ac signal in the genic region, with peak strength defined as the normalized H3K27ac signal per million, subtracting the corresponding input. **(C)** Metaplots of the liver H3K4me3 signal in the genic region, with peak strength defined as the normalized H3K4me3 signal per million, subtracting the corresponding input. **(D)** Overlap of liver ACRs from different pig breeds. **(E)** Stacked bar plot showing the distribution genome features overlapped with ATAC-seq, H3K27ac, and H3K4me3 in EB pigs.

We hypothesized that differences in CREs contribute to phenotypic variation between Chinese and Western pigs. We first assessed chromatin accessibility across tissues (skeletal muscle, liver, and fat) by ATAC-seq from 2-week-old piglets (Zhao et al., 2021). Spearman correlation analysis revealed strong consistency (R = 0.81 ∼ 0.96) among biological replicates of ATAC-seq libraries. The biological replicates from the same tissue and breed were pooled, yielding 84–295 million high-quality mapping reads (Q > 30) per sample (Supplementary Table S1), which provided sufficient data to identify enriched regions (Zhao et al., 2021). The analysis of read distribution around the genic region indicated that the enrichment was proximal to TSSs (Figure 1A; Supplementary Figure S1), with less presence near transcription termination sites (TTSs). Furthermore, H3K27ac and H3K4me3 marks were enriched within 200 bp of TSSs (Figures 1B, C). Using peak-calling software MACS2 (version 2.2.7.1) (Zhang et al., 2008), we identified 71,986–106,686 accessible chromatin regions (ACRs) in Chinese pigs and 55,822–111,990 in Western pigs. To create a comprehensive map of accessible regions across tissues, we merged enriched regions from all samples. This produced a union set of 219,746 ACRs (Supplementary Table S2) with an average length of 740 bp, covering around 6% of the pig genome. Over 42% of these ACRs were shared within specific tissues: 42.2% in the liver (45,810 core liver peaks out of 108,434), 42.5% in fat (59,286 core fat peaks out of 139,531), and 47.8% in muscle (57,940 core muscle peaks out of 121,175) (Figure 1D; Supplementary Figure S2). However, only 13.4% of the union peaks (29,412 out of 219,746) were consistently accessible across all tissues, reflecting significant variations in chromatin accessibility between tissues. The genome-wide chromatin accessibility profiles across tissues were analyzed using pairwise Spearman correlation. We observed a high Spearman correlation (0.88–0.96; see Supplementary Figure S3) between samples from the same tissue but different pig breeds, indicating that inter-breed group variation has minimal impact on chromatin openness. Using the same merging method, we identified 174,640 H3K27ac peaks and 38,500 H3K4me3 peaks activated in one or more tissues (muscle, fat, and liver). Among these, 112,268 H3K27ac peaks (64.3%) and 34,065 H3K4me3 peaks (88.5%) overlapped with ACRs by at least 1 bp.

The pig genome was classified into seven regions: promoter, 5′ untranslated region (5′UTR), coding exon, intron, 3′UTR, downstream, and intergenic regions. Most ACRs and H3K27ac sites were mapped to intergenic regions (88.1%–89.6% for ACRs and 86.5%–88.4% for H3K27ac), followed by introns (5.6%–7.3% for ACRs and 5.3%–7.9% for H3K27ac) and promoter regions (1.9%–5.2% for ACRs and 3.0%–6.6% for H3K27ac) (Figure 1E; Supplementary Figure S4). H3K4me3 was predominantly found in intergenic and promoter regions (Figure 1E; Supplementary Figure S4). A few peaks were identified in the 3′UTR, consistent with previous reports (Makiko et al., 2016). The distribution of ACRs indicates that most cis-regulatory regions in the pig genome are in enhancer and promoter regions. The distribution patterns of H3K4me3 and H3K27ac suggest distinct regulatory roles for these two modifications.

### Variations in chromatin accessibility profiles between Chinese and Western pigs

**FIGURE 2:**
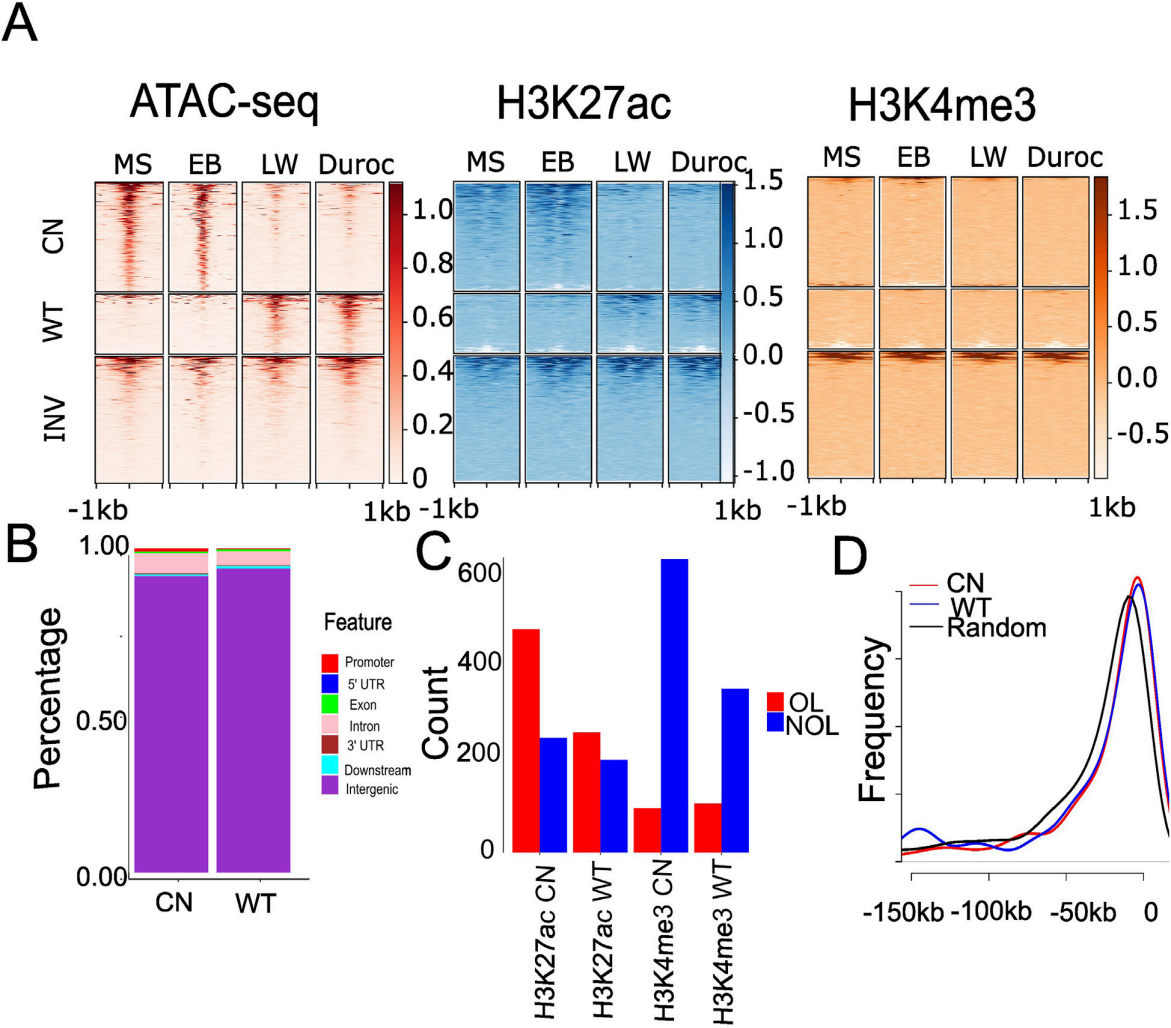
Differential accessibility signals between Chinese and Western gain ACRs in the liver and their overlap with genomic features. **(A)** Heatmap depicting normalized read counts from ATAC-seq, H3K27ac, and H3K4me3 signals in liver tissue across the four pig breeds. Each row corresponds to an ACR, with darker colors indicating increased chromatin accessibility and lighter colors indicating decreased accessibility. “CN” represents Chinese gain ACRs, “WT” denotes Western gain ACRs, and “INV” denotes randomly selected invariant ACRs. **(B)** Stacked bar plot showing the distribution genome features overlapped with significantly changed ACRs. “CN” represents Chinese gain ACRs, and “WT” denotes Western gain ACRs. **(C)** Significantly changed ACRs overlapping with H3K4me3 and H3K27ac. “CN” represents Chinese gain ACRs, “WT” denotes Western gain ACRs, “OL” indicates ACRs overlapping with H3K4me3 or H3K27ac by at least 1 bp, and “NOL” refers to ACRs with no overlap. **(D)** Distribution of distances to the nearest genes for significantly altered ACRs. “CN” denotes Chinese gain ACRs, “WT” represents Western gain ACRs, and “Random” refers to randomly selected invariant ACRs.

We calculated the normalized read count for each ACR to investigate the differences in cis-regulatory elements between Chinese and Western pigs during domestication. DESeq2 was used to identify similarly or differentially accessible regions in pigs. We identified 463 significantly changed ACRs in fat, 454 in muscle, and 1,211 in the liver (false discovery rate (FDR) < 0.05 and |log2FC| >1; Supplementary Table S3). We discovered 743 ACRs (690 from autosomes) that were more accessible in the liver of Chinese pigs, referred to as “Chinese gain ACRs,” and 468 ACRs (409 from autosomes) that were more accessible in Western pigs, referred to as “Western gain ACRs” (Supplementary Table S3). Compared to the known regulatory regions from the FAANG project dataset (Sus_scrofa, Sscrofa11.1.regulatory_features.v112.gff3), Western gain ACRs were more enriched in FAANG regulatory elements, with 67% of Western gain ACRs overlapping these elements versus 57% of Chinese gain ACRs. This difference is statistically significant (P = 0.0002337 by Chi-square test). The fold changes and average read density are shown in Supplementary Figure S5. The Chinese gain ACRs in the liver were more accessible in Chinese pigs (Figure 2A) than in Western ones. However, Western gain ACRs largely became inaccessible in Chinese pigs (Figure 2A). All these regions exhibited partial H3K27ac signals but limited H3K4me3 hits (Figure 2A). The GO term is linked to ACRs according to their proximity to nearby genes. Chinese gain ACRs were found to be involved in signal transduction in fat, cytosolic processes in muscle, and liver energy metabolism activities such as protein binding and guanyl-nucleotide exchange factor activity. In contrast, only oxidoreductase activity was found in muscle tissue in Western pigs, and there was no enrichment of GO terms in fat or liver tissues. Compared to muscle and fat, more differential ACRs were discovered in the liver tissue, suggesting that the accessibility of the liver varied more significantly across the two pig ecotypes. Therefore, all subsequent work focused on liver tissue. The genomic distribution of the gain ACRs was similar (Figure 2B; Wilcoxon rank sum test P = 0.85), which was found to be more abundant (>91%) in intergenic areas. This finding suggests that these regions may serve as distant regulatory components or enhancers. We further explored the relationship between H3K27ac and the gain ACRs, given that H3K27ac signals potentially enhance activity in intergenic regions. The Chinese gain ACRs demonstrated a higher overlap with H3K27ac (66.1%; 491 out of 743, Figure 2C) than Western pigs (56.4%; 264 out of 468, P = 0.0008934 by Chi-squared test, Figure 2C). However, only 13.2% of gain ACRs in Chinese pigs and 23.1% in Western pigs overlapped with H3K4me3 signals (Chi-squared test P = 1.184e-05, Figure 2C). We also investigated the proximity of the gain ACRs to the nearest genes. Most of the intergenic gain ACRs were located within 50 kb of the nearest genes. There was no significant difference between Chinese and Western gain ACRs (Kolmogorov–Smirnov test P = 0.23, Figure 2D), while invariant ACRs exhibited a different pattern (Kolmogorov–Smirnov test P < 4.595e-06). Compared to invariant ACRs, gain ACRs are closer to their target genes, which may facilitate precise and efficient regulation, such as establishing and maintaining tissue-specific gene expression patterns.

### Relationship between gain ACRs and tissue-specific peaks

**FIGURE 3:**
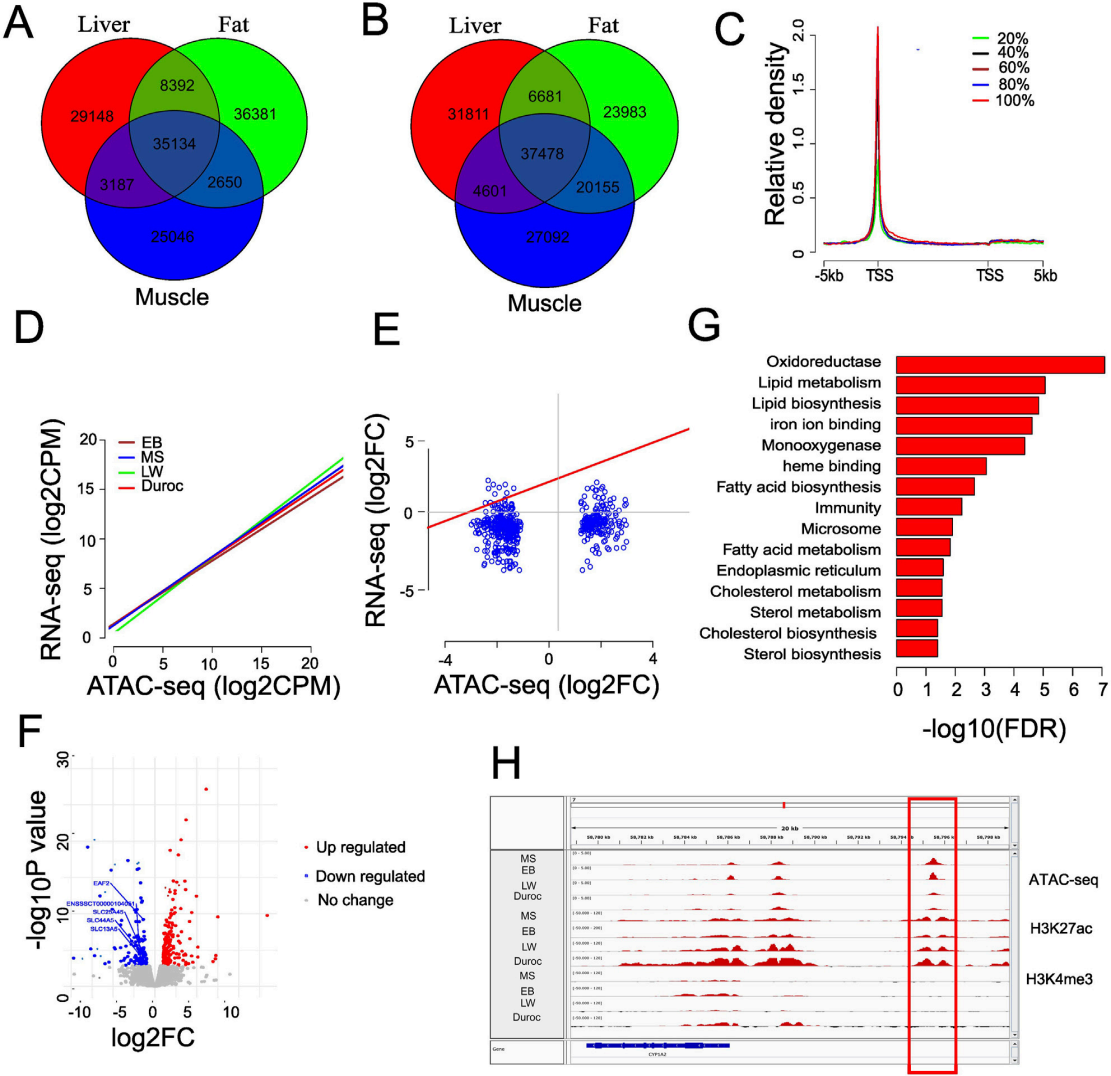
Chromatin accessibility associated with gene expression changes. **(A)** Venn diagram showing tissue-specific peaks in EB pigs by comparing the peaks from the liver, fat, and muscle. **(B)** Venn diagram showing the tissue-specific peaks in Duroc pigs by comparing the peaks from the liver, fat, and muscle. **(C)** Average ATAC-seq profiles in EB liver for genes classified into five equal groups based on liver expression levels. **(D)** Correlation between ATAC-seq signal at fine scale (500 bp surrounding the TSS) and gene expression level in different breeds of pig. **(E)** Connection between nearest gene’s fold alterations and dynamic accessibility variations in liver tissue. **(F)** Volcano graphic showing distinct gene expression patterns in the liver tissue of Chinese and Western pigs. The genes involved in membrane transportation are represented in the text. **(G)** Functional enrichment terms in upregulated genes of Chinese pigs; the resulting GO terms with an FDR value of 0.05 or less were kept. **(H)** Representative enhancer linked to increased gene expression in Chinese pigs with greater chromatin accessibility. ATAC-seq and H3K27ac colocalize but not H3K4me3.

To investigate whether the gain ACRs were preferentially enriched in tissue-specific areas, we compared peaks identified in liver, muscle, and fat tissues from EB and Duroc pigs. EB and Duroc were selected due to their higher number of peaks. We identified 29,184, 25,046, and 36,831 tissue-specific peaks in liver, muscle, and fat, respectively, for EB pigs, 35,154 of them shared across all tissues (Figures 3A, B). In Duroc pigs, we found 31,811, 27,092, and 23,983 tissue-specific peaks in liver, muscle, and fat, respectively, with 37,478 peaks shared across all tissues. Comparing the gain ACRs with EB liver-specific peaks, we found that 448 out of 743 (60.3%) Chinese ACR gains overlapped with liver-specific peaks, contrasting with only 30 out of 468 (6.4%) Western ACR gains. Conversely, 112 (15.1%) Chinese ACR gains overlapped with Duroc liver-specific peaks, while the proportion increased to 52.6% for Western ACR gains. This finding suggests that the gain ACRs were enriched in liver-specific peaks with a background-specific context, and the proportion was higher than peaks from shared tissues (14.5%, Chi-squared test, P = 1.254318e-41). To investigate the efficiency of identifying tissue-specific enhancers by H3K27ac, we categorized the 743 Chinese and 468 Western gain ACRs into 448 (Chinese) and 306 (Western) liver-specific peaks, respectively, and 295 and 162 gain ACRs shared across multiple tissues. Interestingly, the H3K27ac signal level was significantly over-enriched in potential liver-specific enhancers (58.5% vs. 49.2%; P < 0.05 by Chi-squared test) in Chinese gain ACRs. Similar findings were observed for Western gain ACRs (65.4 vs. 33.3; P = 7.613938e-12 by Chi-squared test). The distance to TSS differed significantly between tissue-specific peaks and peaks shared by multiple tissues for Western gain ACRs (Kolmogorov–Smirnov test, P = 0.0165). These results underscored the functional divergence of the gain ACRs across different breeds.

### Chromatin accessibility associated with gene regulation

To explore whether the chromatin accessibility changes were associated with gene expression, we analyzed RNA-seq data from liver tissue from Zhao et al. (2021). We found comparable numbers of expressed genes (normalized counts >1) in the breeds of MS (21,040), EB (22,098), LW (22,430), and Duroc (21,427). To reduce the bias, we categorized expressed genes into five groups, from the lowest 20% to the highest 20%, according to their expression levels. Our analysis revealed a positive correlation between ATAC-seq signal and gene expression levels (Figure 3C). Additionally, we observed a significant positive correlation (Pearson correlation R = 0.42 ∼ 0.43, P < 2.2e-16) between gene expression and chromatin accessibility specifically around transcript start sites (500 bp around TSS, Figure 3D), indicating that chromatin accessibility is a reliable indicator of gene expression levels. We integrated the fold changes in the gain ACRs with RNA-seq data between Chinese and Western pigs to investigate the effects of putative enhancers (i.e., intergenic and intron ACRs within 50 kb of the closest transcription start site) (Supplementary Table S4). Remarkably, we found a weak positive connection (Pearson correlation = 0.24, P = 2.298e-08) between the expression dynamics of nearby genes (<50 kb) and ACR alterations (Figure 3E). When concentrating only on intergenic ACRs, this pattern persisted (Pearson correlation = 0.26, P = 1.012e-06). This correlation improved when only considering genes with significant expression changes, with a Pearson correlation of 0.36 (P = 2.584e-06). This suggests that the expression dynamics of neighboring genes and the directional changes of enhancers are not well aligned. However, genes with significant expression changes might be influenced by the dynamics of ACRs. Using 14,130 TAD boundaries (40 kb) from pig liver as reported in Foissac et al. 2019, we found that approximately 65% of ACR-gene pairs (<50 kb) were located within the same domain. Comparing transcript levels between Chinese and Western pigs, we identified 207 upregulated differentially expressed genes (DEGs) and 133 downregulated genes (FDR<0.05 and |log2FC| >1; Figure 3F; Supplementary Table S5), indicating that most genes in the liver did not undergo significant changes. In Chinese pigs, the upregulated genes showed enrichment in regulating lipid metabolism and lipid, steroid, and fatty acid biosynthesis (Figure 3G). In contrast, DEGs in Western pigs were linked to cellular membrane transport activities but did not meet the FDR cutoff for significance. No significant differences were observed when we compared the fold changes of genes linked to significantly differential ACRs with the fold changes from the entire genome (Wilcoxon rank sum test P = 0.26, Supplementary Figure S6). Considering both significant changes in chromatin accessibility and gene expression, only 53 genes (15.6% of DEGs) were impacted, with distances to the nearest ACRs less than 50 kb and a |log2FC| greater than 1 (Supplementary Table S6). Among the 29 genes located near Chinese gain ACRs, 26 were upregulated (log2 fold change >1). In comparison, 83.3% of the downregulated genes (20 out of 24) were linked to Western gain ACRs. The dataset showed a similar tendency when reducing the cutoff value of fold change >1.6 (Supplementary Table S6). Thus, chromatin activity variations affected a limited set of nearby genes. A potential enhancer was identified just upstream of CYP1A2 (ENSSSCT00000002129) (Figure 3H), showing a similar mRNA fold change (>2.3) and ATAC-seq fold change (>2.4). This gene is a cytochrome P450 enzyme that plays a role in various biochemical processes, including steroid hormone biosynthesis and heme binding. It can influence hormone levels and has a critical role in oxidative reactions due to its heme group. Significant changes were also observed in two other cytochrome P450 family members, ENSSSCT00000055550 and ENSSSCT00000076670, which have comparable functions. This suggests that enhanced distal chromatin accessibility in the liver of Chinese pigs may be crucial for metabolism and hormone regulation.

### Identifying motifs that are functionally associated with potential enhancers

**FIGURE 4:**
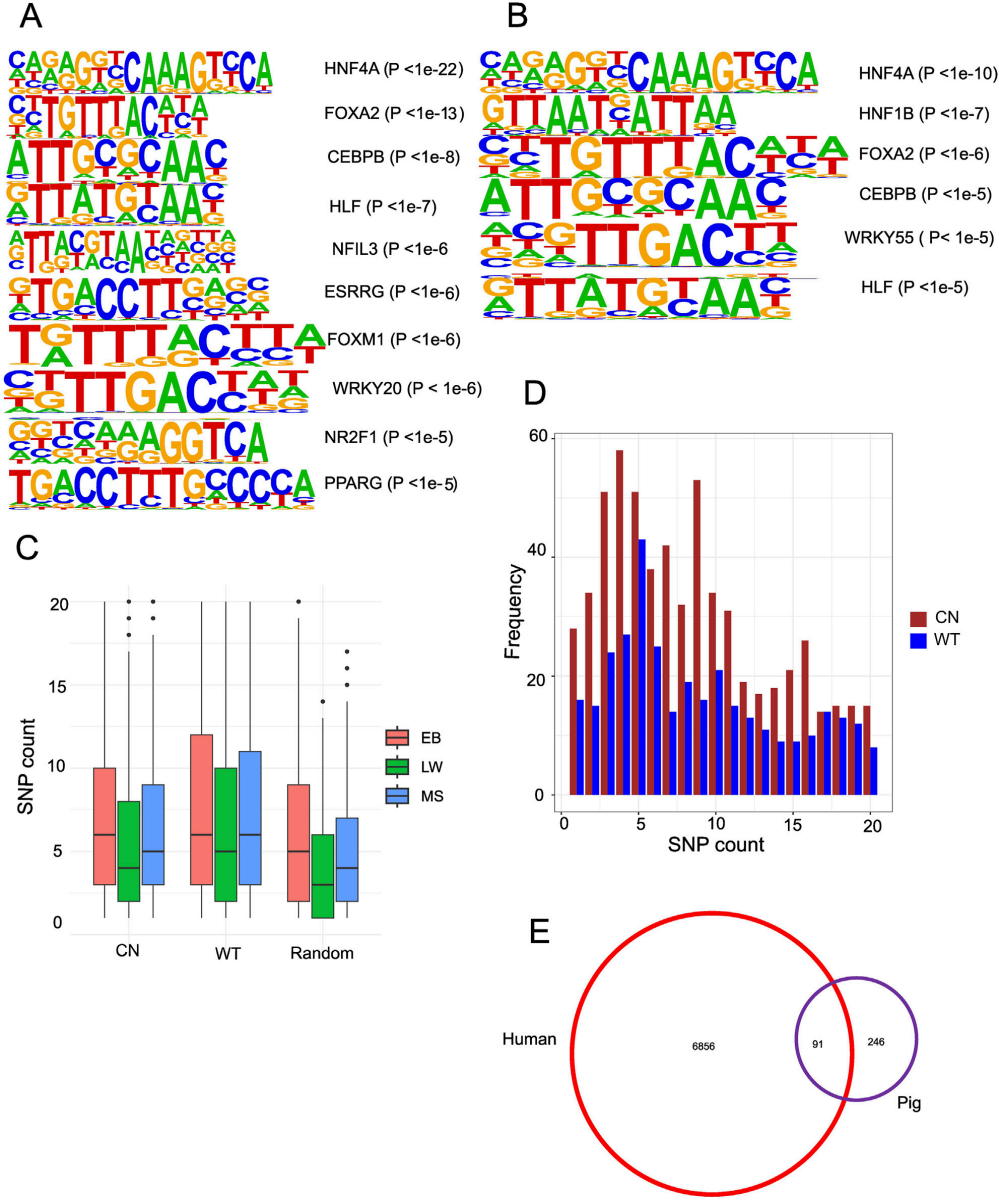
Accessible chromatin regions associated with genetic variation. **(A)** Motifs enriched in liver-specific Chinese gain ACRs. **(B)** Motifs enriched in liver-specific Western gain ACRs. **(C)** Boxplot displaying the number of heterozygous SNPs in Chinese gain ACRs, Western gain ACRs, and the genome-wide average. **(D)** Bar graph depicting the frequency distribution of peaks containing 1–20 SNPs among the gain ACRs. **(E)** Venn diagram illustrating the overlap of DEGs from pig liver tissue that are over-represented among *NR2F1* response genes.

Since chromatin accessibility affects the binding of transcription factors (TFs), *de novo* motif profiling of TFs inside the gain ACRs is essential. Chinese gain ACRs revealed enrichment for nuclear receptors (*HNF4A*, *PPARE*, *ESRRG*, and *NR2F1*), members of the basic leucine zipper (bZIP) family *(NFIL3*, *CEBPB*, and *HLF*), and motifs linked to FOX (*FOXA1*, *FOXA2*, *FOXA3*, and *FOXM1*) (Figure 4A). In Western gain ACRs, enriched motifs included *HNF4A*, *FOXA1/2*, *HNF1B*, *CEBPB*, *WRKY55*, and *HLF* (Figure 4B). The similar patterns observed in both datasets for *FOX(A)*, *HNF4A*, *HLF*, and *CEBPB* underscore the pivotal functions of these TFs. The importance of *FOXA* TFs in the regulatory areas was highlighted by their ability to retain accessible nucleosome structures and attract other liver-enriched TFs to functional enhancers (Makiko et al., 2016). It seems that *NR2F1* is only connected to Chinese gain ACRs. Only a few motifs were found in the gain ACRs from fat and muscle tissue (Supplementary Figure S7). When mRNA expression of potential TFs was examined, none displayed any notable changes (|log2FC|> 1 and FDR <0.05, Supplementary Table S8). As transcription initiation relies on the interaction between TFs and their binding sites, genetic variants within CREs may account for diverse expression patterns. Consequently, it makes sense to investigate how DNA polymorphisms inside CREs affect the binding of TFs.

### Potential impacts of DNA variants on chromatin accessibility and TF binding

To examine the functional implications of genetic variations on chromatin accessibility, we conducted DNA polymorphism analysis by aligning whole-genome sequencing data to the pig reference genome and by calling SNPs using the “bcftools mpileup” command. We identified 14.82 million SNPs in EB, 13.38 million in MS, and 8.45 million in LW. The density of SNPs within the gain ACRs was significantly higher than the random ACR regions (Wilcoxon rank sum test, P < 2.2e-16, Figure 4C). Chinese gain ACRs contained 1–40 SNPs, while Western gain ACRs contained 1–55 SNPs (Figure 4D depicts the peak frequency distribution containing 1–20 SNPs), higher than the random ACR regions containing 1–27 SNPs. A study of cavefish has shown that genetic changes in CREs can affect chromatin accessibility (Jaya et al., 2022). To further investigate the effects of genetic variation on transcription factor binding within the gain ACRs, we performed “motifbreakR” (Coetzee et al., 2015) analysis to predict their impacts on affinity. We identified 1,167 SNPs (13% of 8,960 SNPs in Chinese gain ACRs) predicted to significantly affect the binding of 270 transcription factors (TFs). In Western ACRs, 12.7% of the SNPs (854 out of 6,700) were predicted to impact 240 TF motifs. The proportion of Chinese gain ACRs linked to at least one SNP that could affect TF binding was 53.2%, while the proportion in Western pigs was 57.7%. Interestingly, *NR2F1*, a steroid/thyroid hormone receptor superfamily member, emerged as an important TF with altered binding affinity (Supplementary Table S7). Therefore, the genetic changes between Chinese and Western pig breeds may affect regulatory activity and chromatin accessibility by altering TF binding motifs.

### DEGs in pigs are overrepresented among those responsive to NR2F1

**FIGURE 5:**
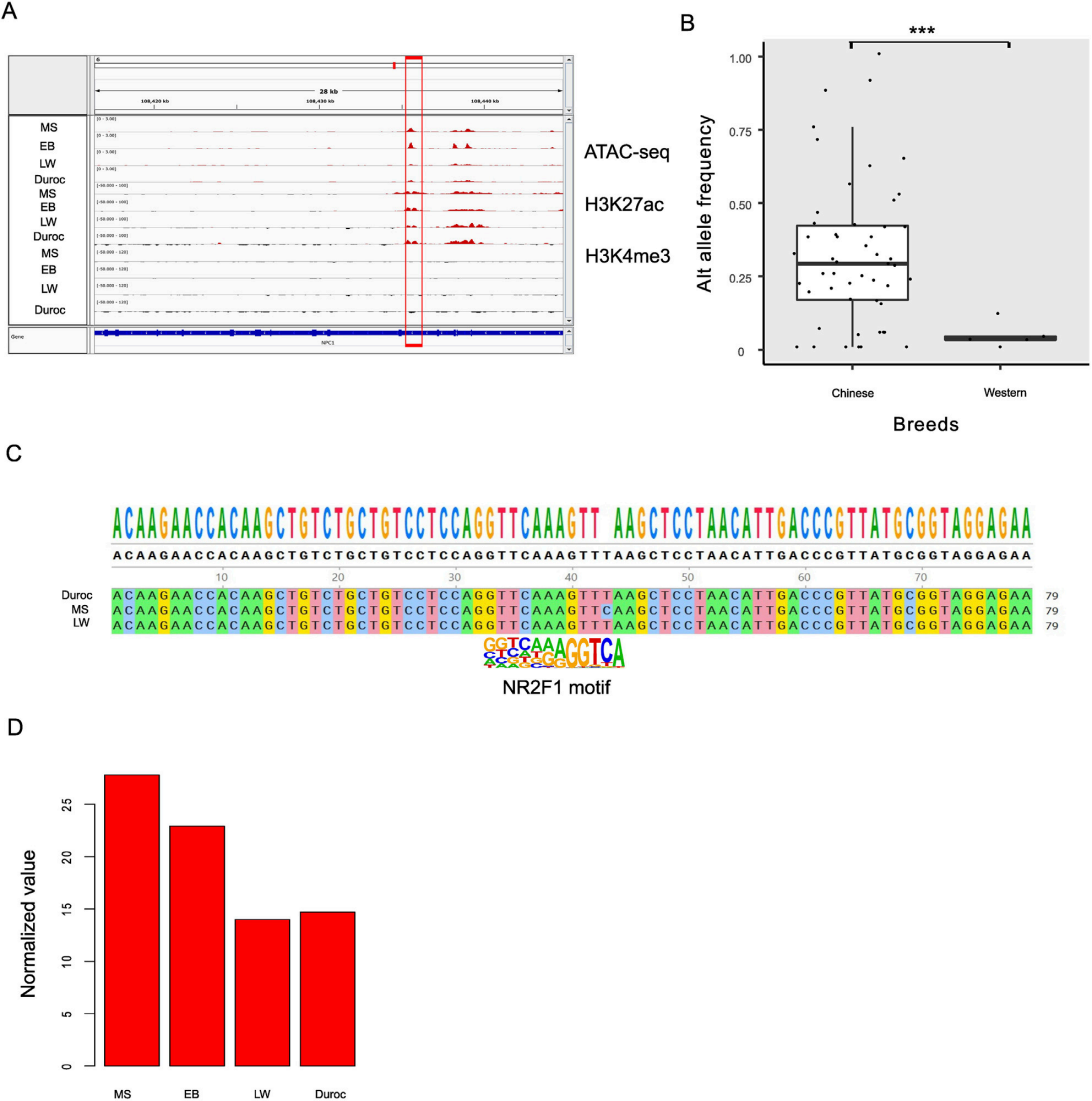
Potential enhancer located in the *NPC1* intron of pigs. **(A)** Dynamic changes in the accessibility of the *NPC1* intron region, along with increased H3K27ac levels in Chinese pigs. The four tracks represent MS, EB, LW, and Duroc breeds. **(B)** Comparison of allele frequencies for the SNP at the 108435531 position on chromosome 6 (Sscrofa11.1, intron of *NPC1*) between Chinese local breeds and Western pigs. **(C)** Performing the genome analysis to verify DNA polymorphisms. **(D)** Normalized expression levels of *NPC1* across pig breeds, with normalization calculated by DESeq2 software.

Identifying the DEGs regulated by *NR2F1* is challenging due to the lack of high-quality antibodies. However, *NR2F1* ChIP-seq data are available for humans. Initially, we compared transcriptome data from two biological replicates of wild-type (WT) and mutant human embryonic stem cell lines (hESCs) containing the *NR2F1-R112K* mutation to discover *NR2F1* response genes (Zhang et al., 2020). A total of 6,947 genes exhibited changed expression levels (FDR <0.05), with slightly more genes upregulated (56%) than downregulated (44%). The DEGs found in pigs were then compared with human results. A total of 15,822 orthologous genes were identified by assigning orthologous gene pairs between humans and pigs based on three criteria: identity >70%, length >500 bp, and E-value < 1e-50. Surprisingly, DEGs in pigs showed significant overlapping with *NR2F1* response genes (91 out of 340 DEGs, P < 0.006, hypergeometric test, Figure 4E). To further explore the role of *NR2F1* activation in Chinese gain ACRs, we identified ten genes closest to Chinese gain ACRs that also overlapped with DEGs in human orthologs. Among these genes, eight showed increased expression levels in Chinese pigs, while two were downregulated (NKD2 and AIF1L). Utilizing publicly available *NR2F1* ChIP-seq data (Zhang et al., 2020) from human cells, we discovered that all eight of the upregulated genes (TIMP3, *NPC1*, RPS6KA2, CYP2D6, SDC1, ABHD2 VTCN1, and TSKU) were directly bound by *NR2F1*, indicating a direct regulatory link between *NR2F1* binding and their expression. Additionally, we found that DNA variation within the intron of the *NPC1* gene was predicted to affect the binding affinity of *NR2F1* in pigs. ATAC-seq data confirmed accessibility in this region, along with H3K27ac enrichment, indicating an active enhancer site within the intronic region of *NPC1* (Figure 5A).

The SNP at position 108435531 on chromosome 6 (Sscrofa11.1, within the intron of *NPC1*) affects *NR2F1* binding affinity and shows a potentially strong selective signal. The reference allele (T) has a frequency of 1 in LW and Duroc but 0 in EB and MS. To examine the alternative genotype fixed in the Chinese population, we analyzed local Chinese populations and compared the results with those from Western pigs. The results showed that the allele frequency of the Chinese and Western pig populations differed significantly (P < 1e-16, Figure 5B). Most Chinese pigs have fixed the alternative allele, especially the Fengjing pig. The Fengjing pig, a native breed from China, shares several positive traits with Taihu pigs, including excellent meat quality, health, and adaptability. However, it grows slowly and accumulates fat. Selection efforts over the years have focused on helping the breed adapt to local environmental conditions and improve its overall fitness while preserving its important traditional characteristics. To avoid false SNP calls from Illumina sequencing, we compared genomes among MS, Duroc, and LW (Figure 5C), finding consistent results. RNA-seq analysis revealed that *NPC1* was 1.77-fold upregulated in Chinese pigs (Figure 5D). This observation suggests that this site may be under positive selection for adapting lipid metabolism and disease resistance in Chinese pigs, although it is strongly selected for breeding purposes in Western pigs.

## Discussion

We conducted a comprehensive genome-wide analysis of the histone marks H3K4me3 and H3K27ac along with ATAC-seq aiming to investigate the regulatory dynamics of chromatin accessibility and the transcriptional landscape differences between Chinese and Western pigs. The dynamic changes in chromatin accessibility have been linked to complex traits and susceptibility to diseases (Mona et al., 2013; Du et al., 2016). Recently, the Functional Annotation of Animal Genomes (FAANG) has been launched to elucidate the regulatory elements in livestock genomes (Giuffra et al., 2018). The divergence in chromatin accessibility between the two subspecies has been studied (Alexandre et al., 2021). However, few studies were conducted on pigs, particularly regarding the mechanisms underlying variations in chromatin accessibility, which remain poorly understood. Here, we investigated the dynamic changes in chromatin accessibility across genetically distant pig breeds to gain insights into how domestication shaped regulatory sequence variations.

Analyzing the genome-wide distribution of H3K27ac and ACRs, we observed that they were most prevalent in the intergenic regions, followed by intronic regions. Over 85% of H3K27ac peaks were found in the intergenic regions, aligning with previous findings that H3K27ac is a marker for active enhancers (Creyghton et al., 2010). Through a comprehensive analysis of ATAC-seq data across pig breeds, we identified numerous chromatin regions that exhibited differential changes between Chinese and Western pigs. Most differentially regulated ACRs were found in liver tissue compared to fat and muscle tissues. Interestingly, 5∼8% of the predicted enhancers were located within intronic regions. Introns are known to play diverse roles in gene expression regulation (Rose, 2019; Shaul, 2017). A recent study indicated that enhancer-like signatures within introns were associated with tissue-specific gene expression (Beatrice et al., 2021). We identified liver-gained ACRs that were predominantly enriched in liver-specific peaks. This suggests that the potential enhancers may play a role in tissue-specific regulation in Chinese pigs.

Regarding gene regulation, we observed a positive relationship between gene expression and chromatin accessibility around the TSS. This conclusion agreed with David et al. (2014), Ampuja. et al. (2017) and Starks et al. (2019). By integrating RNA-seq data with differential accessibility analysis, we found that the gain ACRs were linked to only 15.6% of DEGs, indicating that a notable portion of gene expression changes occurred independently of changes in chromatin accessibility. Additionally, dynamic alterations in chromatin accessibility did not consistently impact the expression levels of neighboring transcripts. This complexity may stem from multiple-layer regulation such as long-distance regulatory mechanisms or poised transcriptional regulation rather than solely focusing on the closest genes. Similar findings of a weak correlation between changes in chromatin accessibility and expression changes have also been reported in geese (Hu et al., 2020). It should be noted that heterogeneity in genetic background or experimental variability among Chinese (EB and MS) and Western (LW and Duroc) pigs could contribute to fewer DEGs. To reduce the false positives and accurately pinpoint genuine genetic distinctions, we implemented a stringent cutoff value (FDR <0.05 and |log2FC| >1). Finally, we identified a few genes which change with chromatin accessibility in the same direction in both pig breeds. For example, the loci with the largest response to accessibility is a gap junction protein, beta 6 (GJB6) with log2 FC > 6 gained in Chinese pigs. Recent studies have suggested that this gene may suppress glycolytic activity through transcriptional mechanisms that have yet to be determined (Jones and Bodenstine, 2022). In Chinese pigs, ATP-binding cassette subfamily member ABCC8 exhibited a more than 16-fold upregulation, crucially linking intracellular glucose metabolism with the exocytosis of insulin-containing secretory granules (Ashcroft, 2005).

To identify TFs relevant to ACRs, we analyzed the differentially changed ACRs to pinpoint potential breed-specific cis-regulatory motifs and their associated TF binding sites. We observed significant enrichment of several well-known liver metabolic regulators, including HNF4A, CEBPB, and FOXAs, in both gained ACRs. FOXA1/2, known not only for their roles as essential competence factors during liver development but also for maintaining crucial hepatic functions in adults, was particularly noteworthy (Yitzhak et al., 2020). HNF4A exhibited liver-specific expression and governed numerous target genes crucial for hepatocyte differentiation, as well as the regulation of postnatal glucose, lipid, cholesterol, and bile acid homeostasis (Kenichi et al., 2020; Fereshteh et al., 2003; Hayhurst et al., 2001). CEBPB was also a TF regulating daily liver metabolism in the liver (Greco et al., 2021). Thus, these members collectively play pivotal roles in liver regulation. In this study, we observed an enrichment of the binding motif for the transcription factor NR2F and its target genes in Chinese pigs. *NR2F1* belongs to the superfamily of transcription factors, possessing both a DNA-binding and a ligand-binding domain, and it plays critical roles in various brain developmental processes. These include the proliferation and differentiation of neural progenitors and the migration and acquisition of identity in neocortical neurons (Tocco et al., 2021). Currently, there is no high-quality *NR2F1* antibody available for use in pigs. However, the high degree of homology (>92%) of *NR2F1* between humans and pigs makes humans a suitable model for investigating its binding targets on a genome-wide scale. We employed human *NR2F1* ChIP-seq data to demonstrate that *NR2F1* acts as a transcriptional activator for numerous target genes within gain ACRs in Chinese pigs. Exploring chromatin-opening mechanisms regulated by *NR2F1* could offer new insights into genome dynamics that evolve during domestication. One of *NR2F1’s* targets, *NPC1*, is a multi-spanning membrane protein known for its role in regulating cholesterol and fatty acid transport in humans (Piyali et al., 2020). A *NPC1* knockout mouse model results in impaired insulin signaling, elevated glucose levels, increased total cholesterol levels, and larger heterogeneous HDL particles (Lamri et al., 2018). *NPC1* has been identified as a potential candidate gene associated with polygenic obesity (David et al., 2009), and further studies found that it was significantly associated with other metabolic traits in human and mouse models (Ruixin et al., 2017). *NPC1* is essential for the endosomal trafficking of virions, facilitating their exit into the cytoplasm during the cell entry process in pigs (Cuesta-Geijo et al., 2022). High polymorphism has been found in this gene in Chinese local breeds, which may influence transcription factor binding and contribute to distinctive traits such as improved meat quality and disease resistance. Collectively, our findings highlight the effectiveness of this integrated genomic approach in characterizing *NPC1* regulation in liver tissue, shaping the unique characteristics observed in Chinese local breeds. This study addresses crucial gaps by identifying and characterizing the function of noncoding regulatory elements, offering an effective strategy to pinpoint candidate loci implicated in lipid metabolism and disease resistance. Further analyses are necessary to elucidate the detailed regulatory mechanisms involved.

## Materials and methods

### Data sources

Four domestic pig breeds—Chinese local breeds Meishan (MS) and Enshi Black (EB) and Western commercial breeds Duroc and Large White (LW) —were selected for cis-regulatory domestic analysis. ChIP-seq analyses for H3K27ac and H3K4me3, as well as ATAC-seq, were performed on skeletal muscle, liver, and fat tissues from the four breeds. Detailed information on sample preparation and library construction is available in Zhao et al. (2021). The human RNA-seq data were from embryonic stem cell lines with *NR2F1-R112K* mutation and wild type (Bolger et al., 2014). *NR2F1-R112K* mutation is a point mutation in the *NR2F1* gene (GRCh38.p12; chr5:93585358G>A; c.335G>A; p.R112K). This mutation results in excitatory and inhibitory neuron imbalance (Makiko et al., 2016), and further research is needed to explore its potential role and mechanisms in neurodevelopmental disorders. The *NR2F1* ChIP-seq data were obtained from wild-type samples and compared with the corresponding input files. The Illumina raw reads for EB, MS, and LW were retrieved from the NCBI SRA database for SNP calling. Detailed information is available in Supplementary Table S1.

### ChIP-seq data processing

Reads were trimmed using Trimmomatic v 0.39 (Bolger et al., 2014), and the trimmed reads were aligned to Sscrofa11.1 pig reference genome (Warr et al., 2020) using BWA-MEM v0.7.17 (Li and Durbin, 2009). We filtered the mapped reads to include only those with a mapping score >30 and sorted them using SAMtools (version 1.15.1). PCR duplicates generated during library construction were removed using MarkDuplicates from Picard Tools (version 2.26.1) (https://github.com/broadinstitute/picard). The Spearman correlation coefficient between two biological replicates was calculated using 10-kb non-overlapping windows. MACS2 (version 2.2.7.1) software was used for peak calling (Zhang et al., 2008). For ATAC-seq analysis, the BAM file was transformed into BED format by bedtools (version 2.29.2) “bamtobed” command. The parameters for peak calling were set as “-g 2e9 -q 0.01 --keep-dup all -f BED --shift -100 --extsize 200”. The peak union method was applied to merge peaks from different tissues and breeds. This approach creates a consensus peak set by merging overlapping peaks from individual tissues into a single, larger peak. This peak merging approach is implemented using the bedtools “merge” command with default parameters. Detailed peak information can be found in Supplementary Table S2. For H3K4me3 and H3K27ac, the parameters used were “-g 2e9 -q 0.01 --keep-dup all -f BAMPE”. The union peaks for H3K27ac and H3K4me3 were created using the same method applied to the ATAC-seq data. For human *NR2F1* ChIP-seq, the parameters were “-g hs -q 0.05 --keep-dup all -f BAMPE”, consistent with those used in Zhang et al. (2020). The “bamCompare” command from Deeptools (Fidel et al., 2016) was used to generate bigwig files for H3K4me3 and H3K27ac, with the parameters “--binSize 20 --operation subtract--normalizeUsing CPM -p 5 --smoothLength 60”. The bamCoverage function from Deeptools (Fidel et al., 2016) was used for ATAC-seq analysis. The density plot was created using the computeMatrix command in Deeptools and then plotted using R.

### Peak annotation analysis

Peaks were annotated with all genomic features using the assignChromosomeRegion function from the ChIPpeakAnno package (version 3.19; Bioconductor). The precedence was set to c (“Promoters,” “immediateDownstream,” “fiveUTRs,” “threeUTRs,” “Exons,” “Introns”), and the Txdb parameter was specified as “TxDb.Sscrofa.UCSC.susScr11.refGene”.

### Peak overlapping analysis

To explore the relationship between ATAC-seq data and the TSS of nearby genes, a random TSS was used when a gene had multiple transcripts. When multiple ACRs were found within 50 kb of the TSS, the nearest ACR was selected for analysis. The distance of peaks to the TSS was determined using the bedtools closest command (version 2.29.2) (Quinlan and Hall, 2010). This measurement was provided as an absolute value without accounting for whether the peak was up- or downstream. The overlap between H3K27ac, H3K4me3, and ATAC-seq peaks was identified using the bedtools “intersect” function (version 2.29.2) (Quinlan and Hall, 2010), with default parameters that require at least 1 bp of overlap.

### RNA-seq analysis

Adapter trimming was performed using Trimmomatic v 0.39 (Bolger et al., 2014). Reads were aligned to the pig reference genome Sscrofa11.1 (Warr et al., 2020) using STAR software (version 2.7.10a) (Alexander et al., 2012) with --outFilterMultimapNmax 1 --outFilterMismatchNmax 2 --outSAMtype BAM SortedByCoordinate and--quantMode GeneCounts to generate a count table. Genome assembly GRCh38.p13 and the corresponding gene transfer format (GTF) file were used for human transcriptome analysis using the same parameter as pigs. Differential gene expression analysis was performed using DESeq2 (Love et al., 2014), only considering genes that had one count or more reads per million level in at least one sample. DEGs were classified as having an FDR-adjusted p-value <0.05.

### Gene ontology analysis

Gene ontology (GO) enrichment was performed using DAVID (https://davidbioinformatics.nih.gov/) (Glynn et al., 2003). GO terms that had a false discovery rate (FDR) of 0.05 or less were considered significant.

### Identification of differentially accessible regions

To assess the chromatin accessibility changes on a genome-wide scale, bedtools’ “multicov” command (version 2.29.2) (Quinlan and Hall, 2010) was used to count read counts in the union peak regions. DESeq2 (Love et al., 2014) was used for pairwise analysis for differentially accessible regions (|log2(FC)|>1 and FDR <0.05). If the regions showed an average fold change >2 in Chinese pigs, we defined them as Chinese gain accessible chromatin regions (ACRs). In contrast, Western gain ACRs were defined when increasing ATAC-seq signals detected in Western pigs and an average fold change >2.

### Genome variant calling among pig breeds

The raw reads were mapped to the reference genome using the BWA-MEM algorithm (version 0.7.17-r1188) (Li and Durbin, 2009); reads with a mapping quality of less than 30 were removed. Duplicated reads were removed using Picard (version 2.26.1) (https://broadinstitute.github.io/picard). We used the bcftools mpileup for genotype calling (Li, 2011), and only binary alleles were considered here. We discarded sites with insufficient read depth (depth >10).

### Transcription factor motif analysis

We assessed whether Chinese gain ACRs and Western gain ACRs were enriched in known sequence motifs of transcriptional factors. The findMotifsGenome.pl command in HOMER (version 4.11) was used to search for enriched motifs in these two sets of peaks with -size given (Sven et al., 2010). We only considered the motifs that were present in higher proportions than expected by chance (i.e., overrepresented motifs) with P-value ≤ 1e-5. The potential effect of SNPs on TF binding motifs from the HOCOMOCO database was estimated by motifbreakR (Coetzee et al., 2015; Kulakovskiy et al., 2017) with the default setting.

## Data Availability

The original contributions presented in the study are included in the article/Supplementary Material; further inquiries can be directed to the corresponding authors.
