# Supplementary material for: Analysis of cis-regulatory changes underlying phenotype divergence shaped by domestication in pigs
Source: Front Genet. 2024 Nov 8;15:1421859. doi: 10.3389/fgene.2024.1421859 (PMC11581869; doi:10.3389/fgene.2024.1421859)
Supplement: Supplementary file 1 [file Table1.DOCX]

**Supplemental information**

**Supplemental Figure 1**

A Metaplot showing the ATAC-seq, H3K4me3, and H3K27ac signals in the genic regions across various tissues. For ATAC-seq, the y-axis represents the peak signal, measured as the normalized value per million reads. The peak strength for H3K4me3 and H3K27ac is the normalized reads per million, adjusted by subtracting the corresponding input.

**Supplemental Figure 2**

The overlap of ACR regions across various pig breeds and tissues

**Supplemental Figure 3**

The Spearman correlation calculated from ACRs in various tissues and breeds

**Supplemental Figure 4**

The genomic features overlapping with ACRs, as well as peaks from H3K4me3 and H3K27ac, across different pig breeds

**Supplemental Figure 5**

MA plots illustrate the difference between gain ACRs and invariant regions across various tissues

**Supplemental Figure 6**

The boxplot displays the fold changes between genes nearest to gain ACRs and the genome average

**Supplemental Figure 7**

The enrichment of motifs in Chinese gain and Western gain regions across different tissues

**Supplemental Table 1**

The sample information utilized in the current study

**Supplemental Table 2**

The data includes union peak information and normalized read counts from various pig breeds

**Supplemental Table 3**

Information on the gain ACRs from various tissues is provided

**Supplemental Table 4**

Information on ACRs located within 50 kb of the TSS has been provided

**Supplemental Table 5**

Information on differently expressed genes has been reported, with "up" indicating genes that are up-regulated in Chinese pigs and "down" indicating genes that are down-regulated in Chinese pigs

**Supplemental Table 6**

Differentially expressed genes associated with significantly altered ACRs, using a fold change cutoff value greater than 1.6, have been reported

**Supplemental Table 7**

The impact of DNA polymorphism on DNA binding affinity has been documented

**Supplemental Table 8**

The expression levels of enriched motifs for transcription factors
