# Supplementary figures and images for: Analysis of cis-regulatory changes underlying phenotype divergence shaped by domestication in pigs
Source: Front Genet. 2024 Nov 8;15:1421859. doi: 10.3389/fgene.2024.1421859 (PMC11581869; doi:10.3389/fgene.2024.1421859)

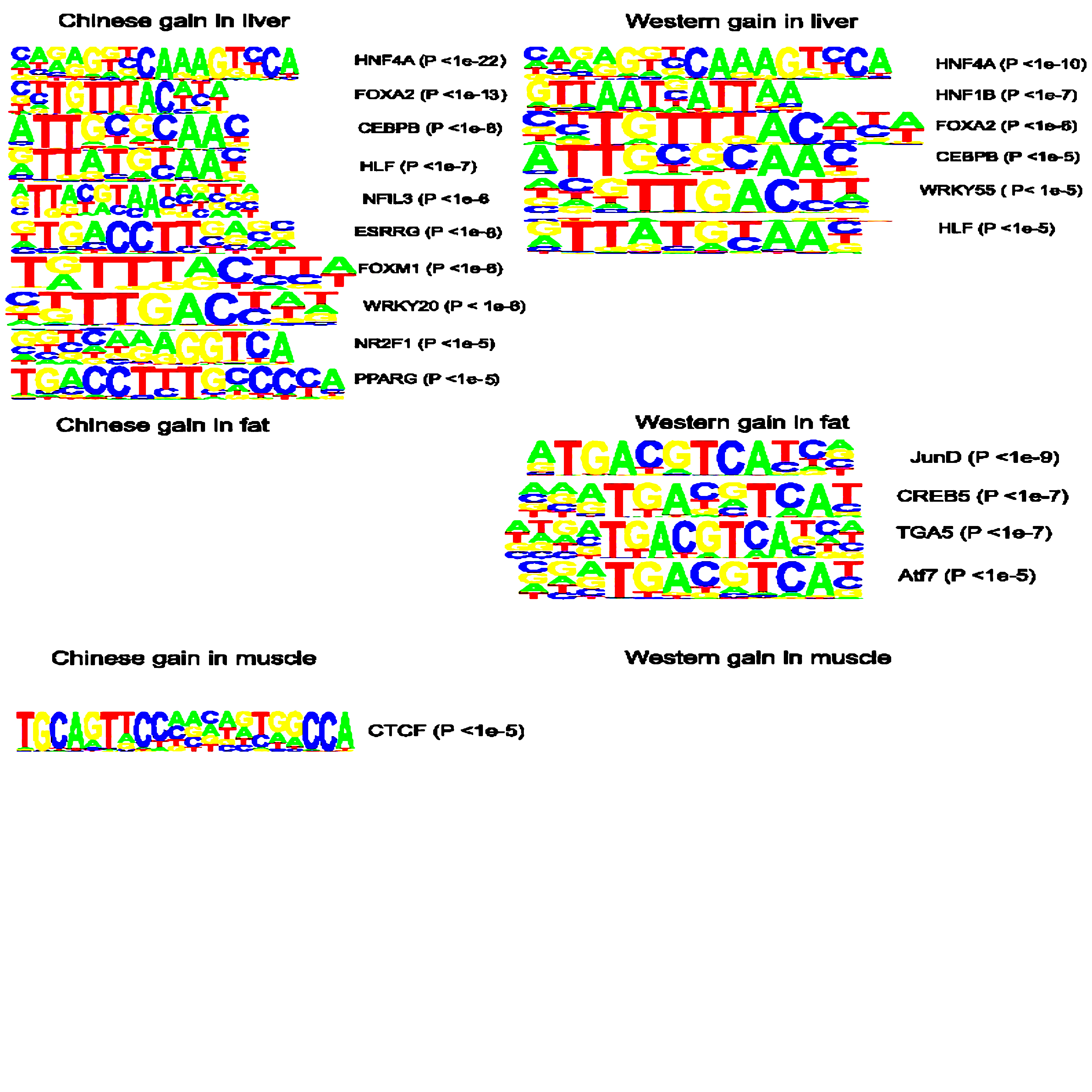

Supplement: Supplementary file 2 [file DataSheet1.ZIP › supplenmentary_figure7.jpg]

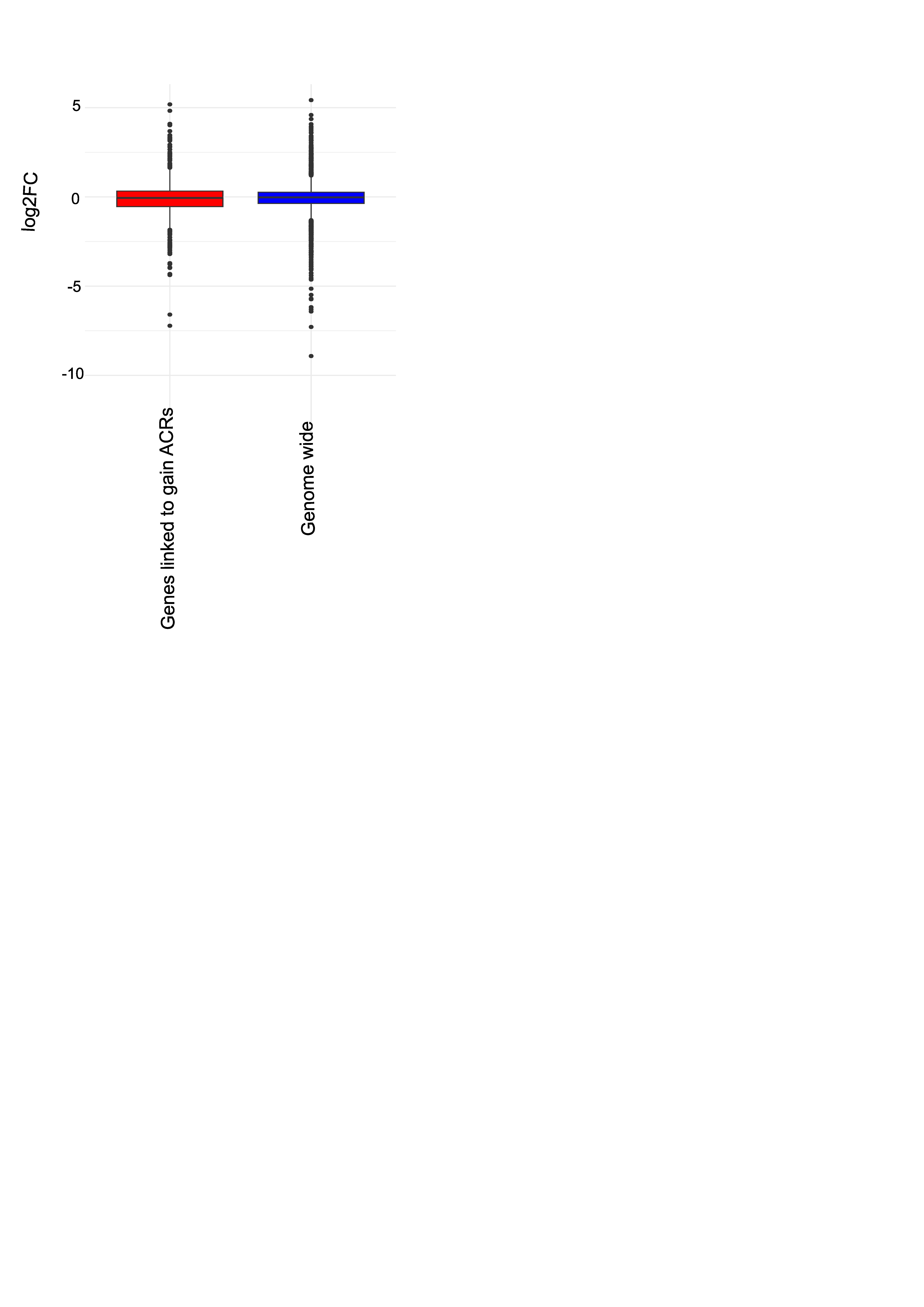

Supplement: Supplementary file 2 [file DataSheet1.ZIP › Supplementary_figure6.jpg]

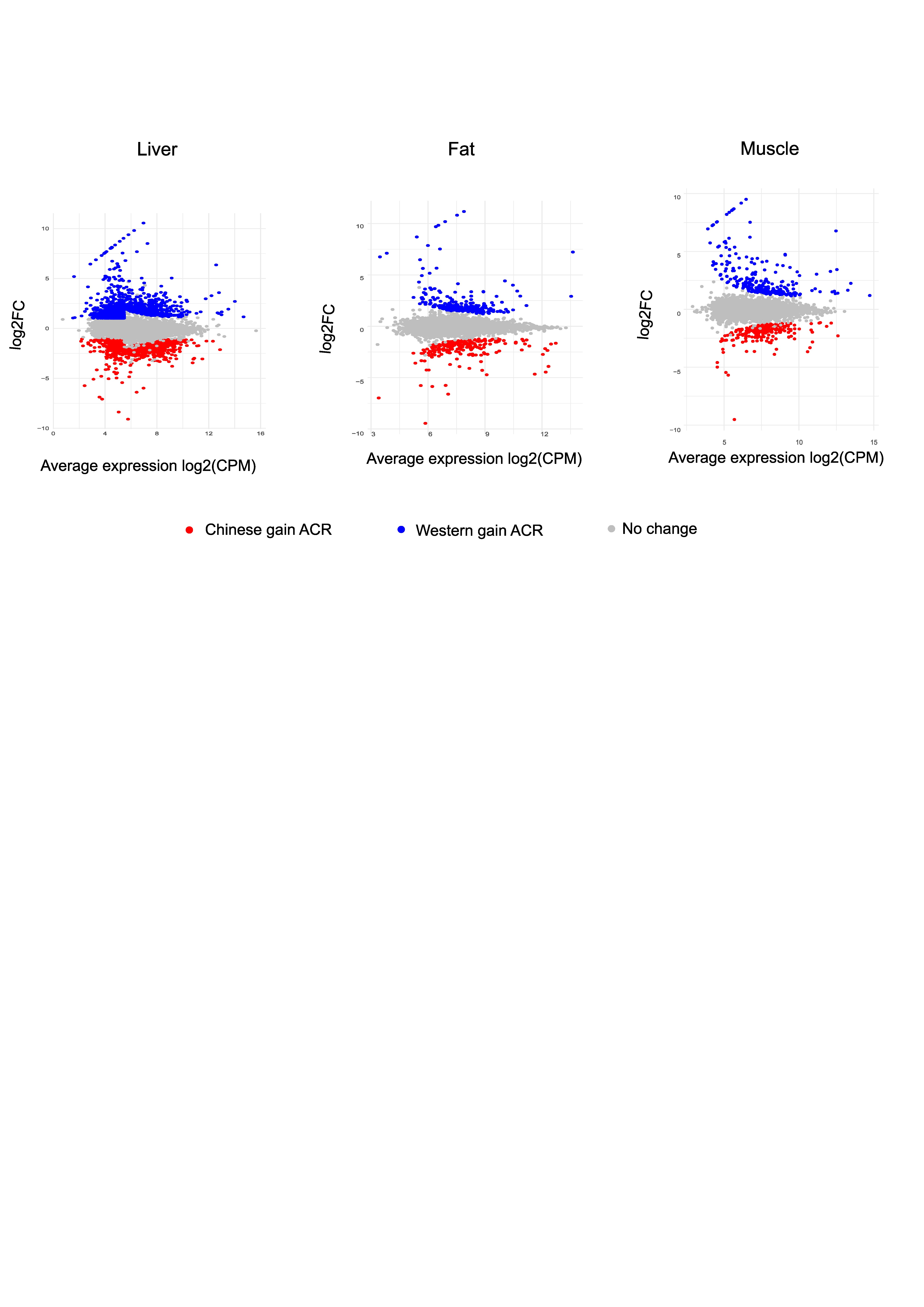

Supplement: Supplementary file 2 [file DataSheet1.ZIP › supplementary_figure5.jpg]

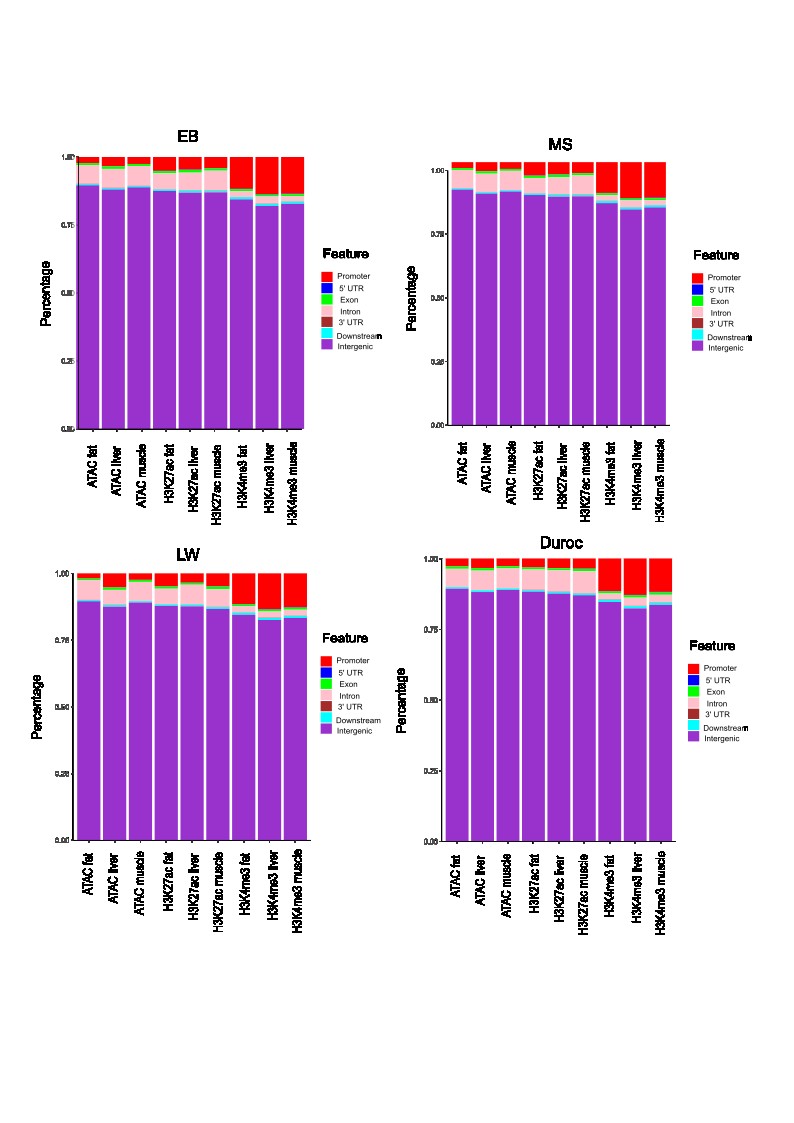

Supplement: Supplementary file 2 [file DataSheet1.ZIP › supplementary_figure4.jpg]

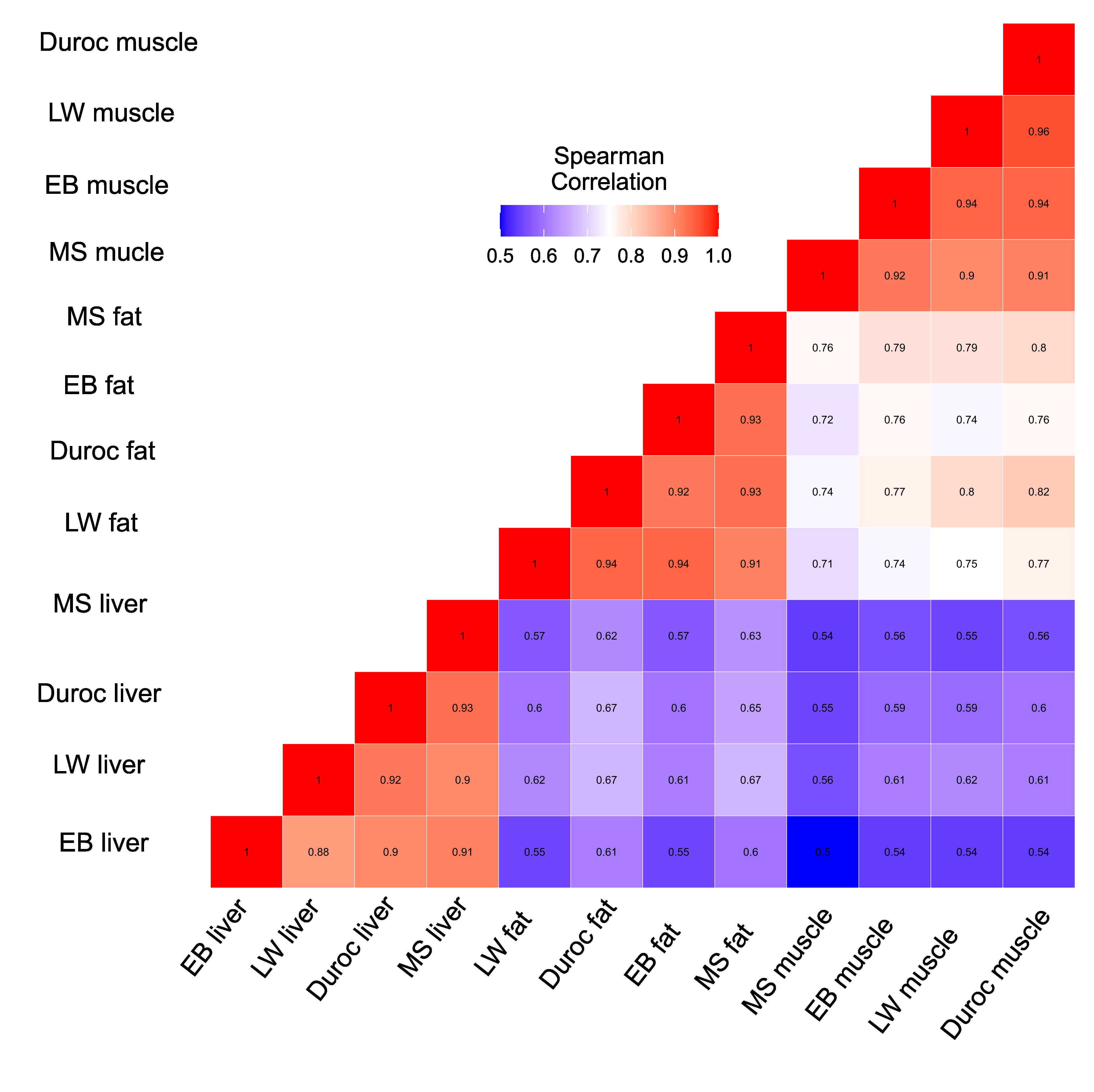

Supplement: Supplementary file 2 [file DataSheet1.ZIP › supplementary_figure3.jpg]

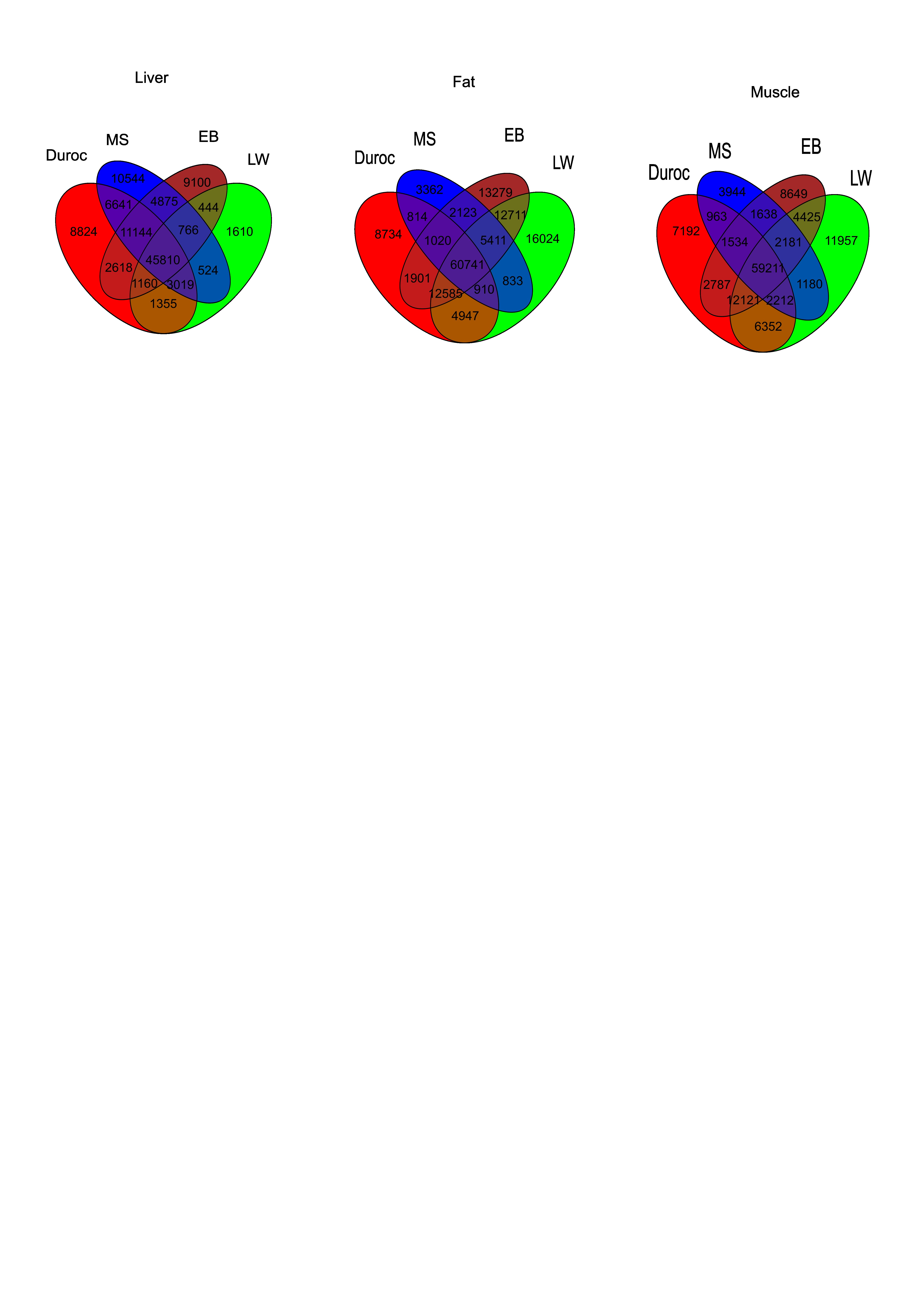

Supplement: Supplementary file 2 [file DataSheet1.ZIP › supplementary_figure2.jpg]

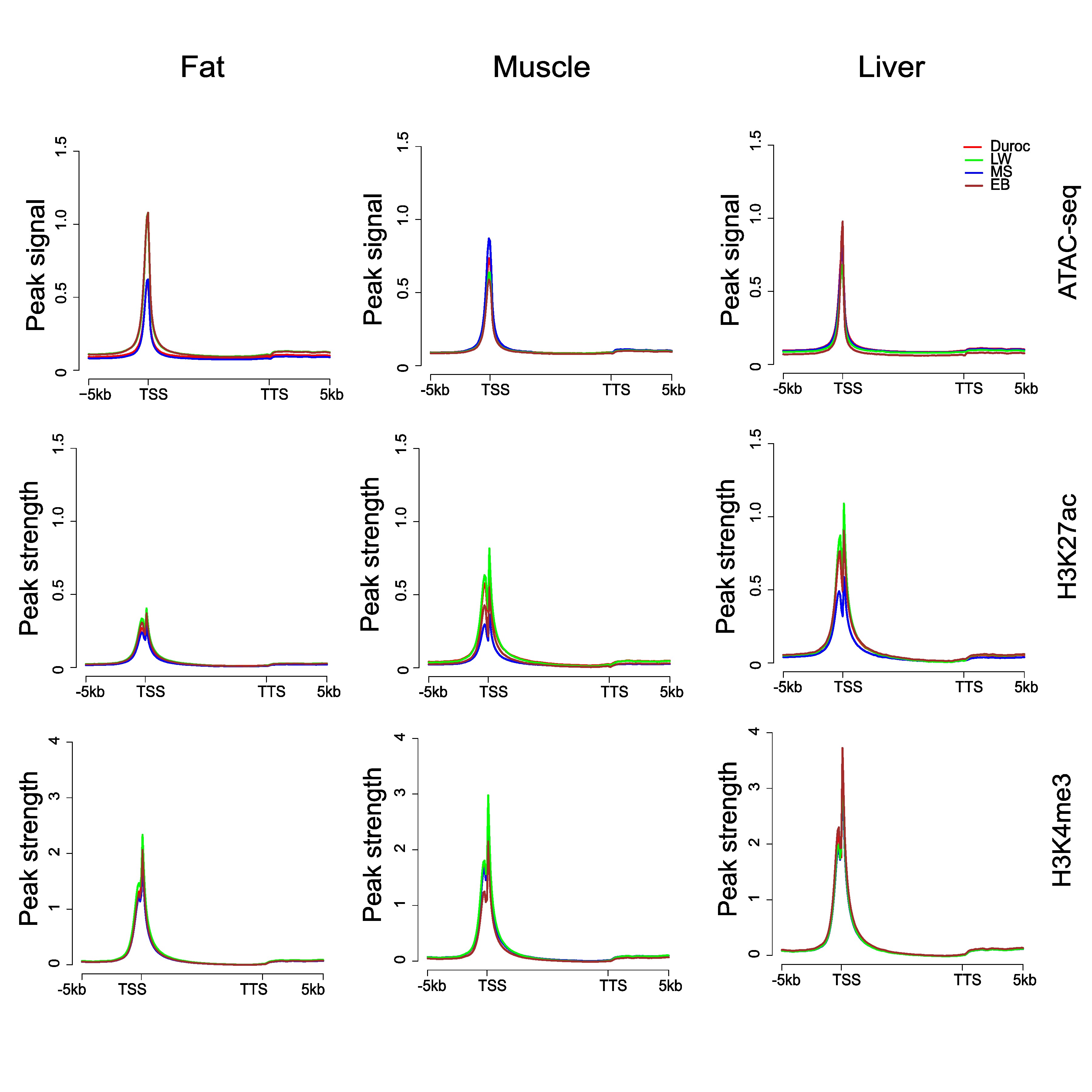

Supplement: Supplementary file 2 [file DataSheet1.ZIP › Supplementary_figure1.jpg]
